# Diagnosis and management of gastroenteropancreatic neuroendocrine neoplasms by nuclear medicine: Update and future perspective

**DOI:** 10.3389/fonc.2022.1061065

**Published:** 2022-11-22

**Authors:** Xing Ma, Ying Ding, Wenliang Li, Qiang Li, Hui Yang

**Affiliations:** Department of Nuclear Medicine, The Affiliated Cancer Hospital of Zhengzhou University & Henan Cancer Hospital, Zhengzhou, China

**Keywords:** nuclear medicine, positron emission tomography, neuroendocrine neoplasms, somatostatin receptor, gastrointestinal cancer

## Abstract

Gastrointestinal (GI) cancers are the second most common cause of cancer related deaths in the World. Neuroendocrine neoplasms (NENs) is a rare tumor that originated from peptidergic neurons and neuroendocrine cells. NENs occurs in all parts of the body, especially in stomach, intestine, pancreas and lung. These rare tumors are challenging to diagnose at earlier stages because of their wide anatomical distribution and complex clinical features. Traditional imaging methods including magnetic resonance imaging (MRI) and computed tomography (CT) are mostly of useful for detection of larger primary tumors that are 1cm in size. A new medical imaging specialty called nuclear medicine uses radioactive substances for both diagnostic and therapeutic purposes. Nuclear medicine imaging relies on the tissue-specific uptake of radiolabeled tracers. Nuclear medicine techniques can easily identify the NENs tissues for their ability to absorb and concentrate amine, precursors, and peptides, whereas the traditional imaging methods are difficult to perform well. The somatostatin receptor (SSTR) is a targetable receptor frequently expressed in the gastroenteropancreatic neuroendocrine neoplasms (GEP-NENs), and is a promising target for tumor-targeted therapies and radiography. SSTR based somatostatin receptor imaging and peptide receptor radionuclide therapy (PRRT) has emerged as a new hot subject in the diagnosis and treatment of GEP-NENs due to the rapid development of somatostatin analogues (SSAs) and radionuclide. This review aims to provide an overview of the current status of nuclear medicine imaging modalities in the imaging of GEP-NENs, and puts them in perspective of clinical practice.

## Introduction

Gastrointestinal (GI) cancers account for approximately 20% of all cancer and are responsible for 23% of cancer-related deaths worldwide (1). The GI epithelial tumors are more common compared to non-epithelial tumors and mainly found in the esophagus, stomach, liver, gallbladder, bile duct, pancreas, small intestine, colon, rectum and the anal region. A subset of GI epithelial lesions exhibits neuroendocrine differentiation and was known as neuroendocrine neoplasms (NENs). Gastroenteropancreatic neuroendocrine neoplasms (GEP-NENs) is one of the most common types of NENs and its incidence has been rising during the past three decades (2, 3). However, the clinical manifestations of NENs are mostly atypical (4). Examination techniques for early diagnosis of GEP-NENs are therefore urgently needed. The timely and accurate diagnosis of GEP-NENs remains challenging for clinicians. Endoscopy and endoscopic ultrasonography (EUS) maybe useful for visualizing tumors found in the stomach, duodenum, rectal and sigmoid. The diagnostic accuracy of traditional imaging techniques, including CT, MR, and US, has improved over the last few years (5, 6). However, traditional imaging techniques are unable to effectively diagnose the small primary tumors which have been metastasized. Additionally, primary midgut tumors that are common in jejunum, ileum, and proximal colon are challenging and difficult to diagnose by gastroscopy or EUS examination. With the extensive use of nuclear medicine in the early diagnosis of GEP-NENs, the clinical benefit for patients has improved widely. Nuclear medicine modalities have the benefit of showing the target tissues’ morphological and functional condition. The tissue origin, cell type, benign or malignant status, level of differentiation, and anatomic placement are used to categorize tumors. Somatostatin receptors (SSTRs) are highly expressed in the GEP-NENs. SSTRs are overexpressed on well-differentiated GEP-NENs tumor cells, especially SSTR2. The exact detection or treatment of GEP-NENs can be accomplished by labeling somatostatin analogues (SSAs) with diagnostic radionuclides or therapeutic radionuclides based on the specificity of the SSTR (7). SSAs or peptide receptor radionuclide therapy (PRRT) can be used to diagnose, stage, and evaluate the effectiveness of the treatment. SSAs and radionuclides have continued to advance, and as a result, nuclear imaging and therapy are currently a popular topic in the field of GEP-NENs.

This review provides an overview of the currently used imaging modalities and ongoing developments in the imaging of GEP-NENs, with the emphasis on nuclear medicine and puts them in perspective of clinical practice.

## Nuclear medical imaging

Routine imaging techniques such as CT and MRI, are the first line anatomic imaging modalities for the diagnosis of NENs. CT/MRI can provide detailed and accurate anatomic information in locating the primary tumor and identifying the local and distant metastases. However, the diagnostic sensitivity of these anatomical imaging techniques is not very high especially in the diagnosis of NENs. Nuclear imaging is a novel imaging technology which combines functional and morphologic techniques (8). This combination can provide more information for the better diagnosis and treatment guidance of NENs. Common nuclear imaging methods for NENs diagnosis include SRS and tumor metabolism imaging (9).

The appropriate use of imaging agents is very crucial for the management of NENs. Gallium-68 SSTR (^68^Ga-SSTR) PET/CT and Fluorine-18-fluorodeoxyglucose (^18^F-FDG) PET/CT are two most commonly used functional imaging for the diagnosis of NENs. Both can localize lesions that are difficult to be detected by traditional imaging techniques, as well as assess the function of the lesion and optimize treatment strategies. Most well-differentiated NENs highly express SSTR (10), which makes it possible to clinically apply SSTR-mediated radiographic imaging, including ^99m^Tc-Octreotide SPECT-CT imaging and ^68^Ga-SSTR PET/CT imaging.

## Diagnostic role of nuclear medicine in GEP-NENs

^18^F-FDG is the most popular molecular probe in nuclear medicine that reflects glucose metabolism *in vivo*. It is more sensitive to tumors with low differentiation and high malignancy, and its absorption and retention are mostly dependent on the expression and phosphorylation level of glucose transporters. According to the Ki-67 index for tumor grading, NEMs are divided into three grades: G1 (less than 2%), G2 (between 22% and 20%), and G3 (more than 20%) (11). Numerous investigations have demonstrated that ^18^F-FDG PET-CT performs poorly in low-grade, well-differentiated GEP-NENs and performs well in high-grade, poorly differentiated GEP-NENs (12, 13). The SUV_max_ of ^18^F-FDG PET/CT and Ki-67 expression are positively correlated as shown in several investigations (14, 15). This finding suggests that ^18^F-FDG PET/CT has an important prognostic value in high grade GEP-NENs. Currently, guidelines published by both European Neuroendocrine Tumor Society (ENETS) and European Association of Nuclear Medicine (EANM) recommend the use of ^18^F-FDG PET/CT to localize high-grade hypodifferentiated GEP-NENs for stratified analysis of patient prognosis prediction using semiquantitative parameters (16).

SSTR is an important target for molecular imaging diagnosis and radionuclide therapy of SSTR-positive tumors (17). GEP-NENs with varying degrees of grading express different amounts of SSTR on their surfaces. ^111^In-Octreotide is the earliest SSTR agonist used in clinical practice and can be used for SPECT-CT, but its low resolution affects its detection of microscopic lesions and metastases (18). Currently, SSTR agonists commonly used in clinical practice include ^68^Ga-DOTATATE, ^68^Ga-DOTATOC and ^68^Ga-DOTANOC (19). ^68^Ga is a positron radionuclide with a half-life of about 67 min. PET/CT imaging using ^68^Ga-labeled SSTR agonists can substantially improve image quality and spatial resolution, compensating for the deficiency of ^111^In-Octreotide (20). ^68^Ga-DOTATATE PET/CT plays an important role in the detection, primary staging, restaging and efficacy assessment of SSTR-positive tumors. The guidelines published by the ENETS and the EANM both recommend the use of SSTR agonist PET/CT as the first-line imaging method for the diagnosis and staging of GEP-NENs.

However, the half-life and positron energy limit the utilization of ^68^Ga SSTR scanning. Poorly differentiated GEP-NENs usually do not express or under-express SSTR, and therefore ^68^Ga-DOTA-SSTR PET imaging is not effective in these tumors. These tumor cells usually have a higher glucose metabolism rate, so ^18^F FDG-PET is more suitable in poorly differentiated GEP-NENs and has a higher sensitivity for metastatic lesions. ^18^F-FDG PET/CT and ^68^Ga-DOTATATE PET/CT have complementary effects. The combined detection of ^18^F-FDG PET/CT and ^68^Ga-DOTATATE PET/CT has better localization and diagnostic value for GEP-NENs than the two alone (21).

## Advances in nuclear medicine diagnosis of GEP-NENs

In recent years, radionuclides with longer half-life and better imaging results have been gradually incorporated into clinical studies with great potential for development. ^64^Cu-DOTATATE is the latest SSTR agonist approved by the FDA for localization of SSTR-positive NENs. ^64^Cu has a longer half-life and better image resolution than conventional radionuclides, and is more sensitive for diagnosing SSTR-positive GEP-NENs. The long half-life of ^64^Cu extends the time window for PET/CT imaging to 3 h, compensating for the shorter half-life of ^68^Ga (22). Another more mature PET imaging agent for NENs is ^18^F-FDOPA (23), which is a structural analogue of dopamine and can reflect the metabolism of dopamine in NENs. However, owing to the difficult synthesis and purification procedure and the early poor yield, it was not generally promoted in clinical practice. Its production has significantly increased in recent years due to improvements in chemical synthesis and labeling processes, and it has been promoted quickly with amazing results. In clinical research conducted abroad, ^18^F-FDOPA has been extensively explored in the neurological, cardiac, and tumor domains, with the tumor study focusing primarily on medullary thyroid cancer and NENs (24). Currently, tumors with low or ambiguous SSTR expression are the key indications for NENs imaging with ^18^F-FDOPA. Piccardo (25) et al. conducted a head-to-head ^18^F-DOPA and SSTR agonists for PET/CT diagnostic meta-analysis, which showed that both examinations could accurately diagnose intestinal NENs, with a combined sensitivity of 95% for ^18^F-DOPA in a lesion-based analysis, slightly higher than the combined sensitivity of 82% for SSTR agonists. Therefore, some studies (26, 27) have recommended ^18^F-DOPA as a second-line screening method as complementary and alternative of SSTR-based imaging agents.

## Therapeutic role of nuclear medicine in GEP-NENs

Surgery is still the preferred treatment for GEP-NENs (28), and systemic chemotherapy is an another option for individuals who are not candidates for surgery. Targeted therapy can be divided into non-radiolabeled SSA and PRRT. SSA has been applied in the clinical practice for more than 20 years. However, SSA has a relatively limited impact on establishing tumor biology and imaging remission, but it can successfully treat the clinical symptoms of hormone overproduction and stop NENs development (29). Since SSTR-targeted PRRT has been utilized in clinical settings in Europe and the US, it has proven to be an effective method for treating NENs and other SSTR-positive cancers, particularly GEP-NENs. PRRT utilizes therapeutic radionuclide ^177^Lu and ^90^Y-labeled SSTR agonists to deliver precise targeted internal radiotherapy to GEP-NENs.

SSA therapy is primarily indicated for the initial treatment of patients with carcinoid syndrome and unresectable tumors. In contrast, PRRT therapy is indicated for patients with SSTR-positive, G1/2 grade advanced GEP-NENs. The second-generation ^90^Y-DOTA-TOC and the third-generation ^177^Lu-DOTATATE are the most commonly used PPRT therapeutics. Several studies now confirm the safety and efficacy of ^177^Lu-DOTATATE-mediated PRRT (30, 31). It is well tolerated by patients, has a low incidence of acute and long-term adverse effects, and is effective in reducing the specific symptoms of neuroendocrine syndrome, such as diarrhea, facial flushing, and cardiac dysfunction caused by right heart failure. In addition, PRRT has a significant analgesic effect, especially for bone pain caused by bone metastases from gastrointestinal or bronchial NENs.

## Therapeutic advances in nuclear medicine of GEP-NENs

The results of combination therapy with PRRT showed the highest response rate for NENs with the combination of ^90^Y-DOTA-TOC and ^177^Lu-DOTA-TATE (38.1%), with a low rate of tumor recurrence and mild adverse effects in most patients (32). There may be additional benefit from receiving a combination of both nuclides (33). The rationale for this treatment modality is to use the shorter tissue penetration of moderate-energy β-rays emitted by ^177^Lu and the longer tissue penetration of high-energy β-rays emitted by ^90^Y to achieve greater killing effect on both smaller and larger tumors when applied in combination.

In PRRT research, the results of animal experiments and clinical trials show that the antagonist probe ^177^Lu-OPS201 has higher tumor radiation dose and better radiation safety than the agonist probe ^177^Lu-DOTATATE, so it is more suitable for PRRT clinical research of NENs (34). Radionuclide labeled SSTR antagonist ^177^Lu-DOTA-JR11 has a higher tumor uptake rate and a longer tumor retention time than SSTR agonist ^177^Lu-DOTA-TOC, thereby increasing the radiation dose in the tumor by 1.7-10.6 times. The tumor growth delay time and the median survival time of patients were prolonged. Other studies have shown that after PRRT treatment of NENs patients with radionuclide labeled SSTR antagonist, it has a high uptake rate in all known lesion sites (liver, lymph node and bone) (35). Albrecht et al. (36) compared the efficacy of two cycles of PRRT with ^177^Lu-DOTA-JR11, an antagonist of radionuclide labeled SSTR, and ^177^Lu-DOTA-TOC, an agonist, in orthotopic NENs xenograft tumor mice. Mice treated with ^177^Lu-DOTA-JR11 had significantly reduced tumor mass and almost no viable remaining tumor tissue 3 weeks after the end of two cycles of PRRT. In addition, the results of preclinical studies have shown that nuclide labeled SSTR antagonists induce more DNA double-strand breaks than agonists, resulting in better therapeutic effects (37). Therefore, it is of great significance to use radionuclide labeled SSTR antagonists as a neoadjuvant tool for PRRT in NENs patients. At present, a series of targeted imaging and therapeutic drugs based on radionuclide labeling are also being developed (38–40). It is believed that there will be major innovations in this field in the near future.

## Conclusion and future perspective

GEP-NENs are challenging to diagnose and localize due to their wide anatomical distribution and complex clinical features. Although traditional techniques (CT, MRI) have significantly advanced during the last two decades, identification and detection of small primary GEP-NENs tumors still remains challenging. The staging and early identification of the disease is also very crucial for selection of the right treatment and effective management of the patients in timely manner. Using radionuclide-labeled SSTR analogues for nuclear medicine imaging of GEP-NENs shows superior imaging sensitivity and specificity as well as prognostic significance. At present, it serves as the gold standard for GEP-NENs diagnosis, localization, and staging. In future, with the improved technology and introduction of new tracers might further improve the sensitivity and specificity of these methods. Currently available and published data on tumor-targeted radioactive therapy is very encouraging. It has been acknowledged that PRRT has a therapeutic benefit in the management of advanced GEP-NENs and that it has significant potential for advancement as a first-line therapy. More individuals can now benefit from PRRT thanks to combination therapy and recent advances in pharmaceuticals. Nuclear medicine is now more useful in the diagnosis and treatment of GEP-NENs as a result of advancements in research on radionuclides and their ligands. However, it has to be further improved both in terms of dosages and patient’s selection. We are aware that debates are still open in this area and will continue in the future. To get at a more compelling consensus, more assessments and thorough clinical investigations are required. However, it is important to stress that the role of nuclear medicine has grown over the last two decades, and its daily practice can confirm that these methods do offer many alternative valid solutions in the field of GEP-NENs diseases, as well as in other diseases.

## Author contributions

HY and XM formulated the concept of this study; XM wrote the original manuscript draft; YD, WLL, QL provided the data and material support; HY and YD critically revised the manuscript. All authors contributed to the article and approved the submitted version.

## Acknowledgments

​We apologize to the authors whose study could not be cited for the limited spaces. We would like to thank Dr. Yang Shi and Dr. Yingying Zhang for their suggestions and comments on this manuscript.

## Conflict of interest

The authors declare that the research was conducted in the absence of any commercial or financial relationships that could be construed as a potential conflict of interest.

## Publisher’s note

All claims expressed in this article are solely those of the authors and do not necessarily represent those of their affiliated organizations, or those of the publisher, the editors and the reviewers. Any product that may be evaluated in this article, or claim that may be made by its manufacturer, is not guaranteed or endorsed by the publisher.
